# Impaired OXPHOS Complex III in Breast Cancer

**DOI:** 10.1371/journal.pone.0023846

**Published:** 2011-08-25

**Authors:** Kjerstin M. Owens, Mariola Kulawiec, Mohamad Mokhtar Desouki, Ayyasamy Vanniarajan, Keshav K. Singh

**Affiliations:** 1 Department of Cancer Genetics, Roswell Park Cancer Institute, Buffalo, New York, United States of America; 2 Fred Hutchinson Cancer Research Center, Seattle, Washington, United States of America; 3 Department of Pathology and Laboratory Medicine, Medical University of South Carolina, Charleston, South Carolina, United States of America; 4 Departments of Genetics, Pathology, Environmental Health, Center for Free Radical Biology, Center for Aging and UAB Comprehensive Cancer Center, University of Alabama at Birmingham, Birmingham, Alabama, United States of America; University of Windsor, Canada

## Abstract

We measured the mitochondrial oxidative phosphorylation (mtOXPHOS) activities of all five complexes and determined the activity and gene expression in detail of the Complex III subunits in human breast cancer cell lines and primary tumors. Our analysis revealed dramatic differences in activity of complex III between normal and aggressive metastatic breast cancer cell lines. Determination of Complex III subunit gene expression identified over expression and co-regulation of UQCRFS1 (encoding RISP protein) and UQCRH (encoding Hinge protein) in 6 out of 9 human breast tumors. Analyses of UQCRFS1/RISP expression in additional matched normal and breast tumors demonstrated an over expression in 14 out of 40 (35%) breast tumors. UQCRFS1/RISP knockdown in breast tumor cell line led to decreased mitochondrial membrane potential as well as a decrease in matrigel invasion. Furthermore, reduced matrigel invasion was mediated by reduced ROS levels coinciding with decreased expression of NADPH oxidase 2, 3, 4 and 5 involved in ROS production. These studies provide direct evidence for contribution of impaired mtOXPHOS Complex III to breast tumorigenesis.

## Introduction

Mitochondria are essential organelles which perform diverse cellular functions, including respiration through oxidative phosphorylation (mtOXPHOS), which proceeds through the coordinated action of 5 inner mitochondrial membrane protein complexes. During mtOXPHOS, sequential oxidation-reduction reactions at complexes I (NADH dehydrogenase), II (succinate dehydrogenase), III (coenzyme Q: cytochrome *c*-oxidoreductase), and IV (cytochrome *c* oxidodase) are coupled to the translocation of protons across the inner mitochondrial membrane. The resulting electrochemical gradient is ultimately utilized by complex V (ATP synthase) for the generation of ATP from ADP and inorganic phosphate [1], [2]. Thirteen of the subunits involved in mtOXPHOS are encoded by the mitochondrial DNA (mtDNA). The remaining subunits (approximately 85 subunits) are encoded by the nuclear DNA and are targeted to the mitochondria by a mitochondrial targeting sequence.

Otto Warburg observed that cancer cells have an irreversible injury to respiration that leads to decreased oxidative phosphorylation (mtOXPHOS) and increased aerobic glycolysis, despite the presence of sufficient oxygen for aerobic respiration [3], [4]. Irreversible injury to respiration suggested by Warburg involves changes at the genetic level. These include alterations in nuclear gene expression or mutations in genes affecting mtOXPHOS. Recent studies have shown that mutations in mtDNA and/or alterations in mtDNA content also underlie the irreversible injury to respiration [5]–[8].

MtOXPHOS complex I-III contains iron-sulfur proteins that aid in the transfer of electrons within the protein complexes. Rieske iron-sulfur protein (RISP) in Complex III that binds [2Fe–2S] cluster *via* an arrangement of 2 histidines and 2 cysteines [9]. RISP has been shown to be the rate limiting step in Complex III activity. This protein has been associated with oncogene-induced senescence [10], however, its role in tumorigenesis is not described. This study examines mtOXPHOS status in breast cancer cells and primary breast tumors and determines a role for complex III in breast tumorigenesis.

## Methods

### Cell culture conditions

All the cell lines were purchased from ATCC (Manassas, VA). MCF12A cells were grown in DMEM F12 50/50 media supplemented with 10% horse serum (MediaTech), 0.1 µg/ml cholera toxin (Sigma, St. Louis, MO), 20.0 ng/ml epidermal growth factor (PeproTech, Rocky Hill, NJ), 0.1% penicillin/streptomycin (MediaTech), 0.5 µg/ml hydrocortisone (Sigma), 1.2 g/L sodium bicarbonate (Sigma) and 10.0 µg/ml insulin (Sigma). All other cell lines were maintained in DMEM (MediaTech) with 10% fetal bovine serum (MediaTech) and 0.1% penicillin/streptomycin. ρ^0^ cells were supplemented with 50 µg/ml uridine (Sigma). All cells were maintained in a 37°C, 95% humidity, and 5% carbon dioxide environment.

### Western blot analysis

Cells were lysed in RIPA lysis buffer (50 mM tris pH 7.4, 150 mM NaCl, 1 mM PMSF, 1 mM EDTA, 1% triton x-100, 1% sodium deoxycholate and 0.1% SDS) (all Sigma), with addition of Protease Inhibitor Cocktail (Roche). Peroxidase labeled anti-mouse and anti-goat IgG (H+L) were used as secondary antibodies (Vector Laboratories, Burlingame, CA). Anti-α-tubulin (Molecular Probes, Eugene, OR) and anti-actin (Santa Cruz Biotechnologies, Santa Cruz, CA) antibodies were used as loading controls. A premixed cocktail containing 5 monoclonal antibodies against subunits of mtOXPHOS complexes (Mitosciences, Eugene, OR) was used to detect a representative subunit from all 5 mtOXPHOS complexes. Anti-RISP antibody was from Molecular Probes.

### Oxidative phosphorylation enzyme activities

Mitochondria were isolated by differential centrifugation in a sucrose gradient as described in O'Malley et al. [11]. Protein concentrations were determined by the Bradford assay. Oxidative phosphorylation enzyme activities were measured on isolated mitochondria as previously described [12]–[14]. All chemicals for the mtOXPHOS enzyme assays were obtained from Sigma. All spectrophotometric measurements were performed in 1-ml cuvettes (1 cm) using Thermo Spectronic Genesis-6 spectrophotometer (Waltham, MA). Individual mtOXPHOS complex activity is expressed as a ratio to MCF12A cells for each individual respiratory chain complex.

### mRNA Expression Level

The study was approved by Roswell Park Cancer Institute Institutional Review Board, permit number I1085M. Consent from patients was not needed, as the anonymous tissue samples were used for study. These samples were collected by the biorepository resource facility of the Roswell Pak Cancer Institute and provided to us under IRB approved permit number I1085M. RNA from breast and normal tissue samples was reverse transcribed using Superscript III first strand kit (Invitrogen). The RNA was isolated from cell lines by Trizol extraction according to the manufacturer's protocol (Invitrogen) and reverse transcribed using the Superscript III First Strand kit (Invitrogen). Complex III mRNA expression was determined using GoTaq Green 2X Master Mix (Progmega, Madison, WI) with a thermocycler reaction with 1 cycle of 96°C for 2 min; 26–28 cycles of 96°C for 30 s, 60°C for 30 s, 72°C for 30 s; 1 cycle of 72°C for 10∶00. Primers for Complex III are listed in table 1. Expression level of NOX1-5 in 143B RISP knock-down clones was measured as described in Graham *et al.* [15].

**Table 1:** Primers for PCR of Complex III subunits.

Gene Name	Primer Sequence	Direction
*UQCRFS1*	5′-GGCAACGGCAGTAATAACCA-3′	Forward
	5′-CCCACACAGACATCAAGGTG-3′	Reverse
*UQCRH*	5′-ACTGGAGGACGAGCAAAAGA-3′	Forward
	5′-TGATGCCCAGATGATGAAGA-3′	Reverse
*CYC1*	5′-CCAAAACCATACCCCAACAG-3′	Forward
	5′-TATGCCAGCTTCCGACTCTT-3′	Reverse
*UCRC*	5′-CTTCAAAGCCCTCTGCAAAC-3′	Forward
	5′-GCCCAAGACAATTCTTCCAA-3′	Reverse
*UQCRQ*	5′-AGTGCAGTGGTGTGATCTCG-3′	Forward
	5′-CTGTGCCCATTTCCTCATCT-3′	Reverse
*UQCR*	5′-GAAACCCACAGCTCAGCTTC-3′	Forward
	5′-AGACTTCTCAGGGTGGCTCA-3′	Reverse
*CYTB*	5′-TGAAACTTCGGCTCACTCCT-3′	Forward
	5′-AATGTATGGGATGGCGGATA-3′	Reverse
*UQCRB*	5′-TTCTCTGTTCGCGATGTGAC-3′	Forward
	5′-GCTGCATCCACAGACTTCAA-3′	Reverse
*UQCRC1*	5′-AATGGGGCAGGCTACTTTTT-3′	Forward
	5′-GGTCAAGTCTGCACGAGACA-3′	Reverse
*UQCRC2*	5′-CAAAGTTGCCCCCAAACTTA-3′	Forward
	5′-AGCCATGTTTTCCCTTGTTG-3′	Reverse

### Immunohistochemistry of RISP protein

The study was approved by Roswell Park Cancer Institute Institutional Review Board, permit number I1085M. Consent from patients was not needed, as the anonymous tissue array slide from the Cooperative Human Tissue Network (CHTN) and Breast Cancer Program 1 of the National Cancer Institute of Health (Bethesda, MD) was used for immunohistochemistry (IHC). The slide contained benign breast tissue (n = 15), Ductal carcinoma *in situ* (DCIS; n = 12), Invasive ductal carcinoma (IDC; n = 13), invasive lobular carcinoma (ILC; n = 7) and metastatic breast carcinoma in lymph nodes (LNM; n = 7). A section from formalin-fixed, paraffin-embedded benign breast tissue slide was used as a positive control, and characterization of the lesions and grading of the tumors was done by a pathologist. The immunohistochemistry protocol described in Desouki *et al*., [16] was applied with modifications. Briefly, the slides were de-paraffinized by incubation in xylene and ascending grades of alcohol. Antigen retrieval was done by heating in citrate-based, antigen unmasking solution for 30 minutes at 98°C, incubated in 3% hydrogen peroxide for 10 minutes, blocked with blocking peptide for 30 minutes, incubated with anti-RISP antibody in a dilution of 1∶100 overnight at room temperature, followed by incubation with secondary anti-mouse antibody for 30 min and another 30 min with the Vectastatin ABC kit (Vector Laboratories). Color was developed by incubating slides with peroxidase substrate solution followed by counterstaining with hematoxylin. Sections from breast carcinoma cases were also incubated with secondary antibody to check for nonspecific binding. All sections were examined with Olympus BX50 microscope. The pictures were taken with an Olympus DP 70 connected to DP Controller software (Olympus, Center Valley, PA). Scoring of immunoreactivity was considered to be negative or positive, with the same parameters we described (score +  =  < 10% positive, score ++  =  10–50% positive and score +++  =  > 50% positive) [16].

### shRNA knock-down of RISP protein

Expression of RISP was knocked-down using short-hairpin RNAs in a retroviral RNAi-Ready pSIREN-RetroQ vector kindly provided by Dr. Navdeep Chandel (Northwestern University Medical School). Two sequences were used for targeting RISP mRNA; RISP #2 5′AAUGCCGUCACCCAGUUCGUU-3′ and RISP #11 5′-CCUACAUCCCGAUCGAUGAUG-3′ [17]. As a control, pSIRN-RetroQ containing an shRNA sequence against *Drosophila* HIF (5′-CCUACAUCCCGAUCGAUGAUG-3′) was used. Viral stocks were produced by transfecting Phoenix cells (293 packaging cells) with the pSIREN vectors using FuGene HD Transfection reagent (Roche). Virus was harvested 24 and 48 h after transfection. Cells were transduced with the virus in the presence of 4 µg/ml polybrene (Sigma) and stably transduced cells were selected for in puromycin (Sigma).

### Mitochondrial membrane potential and ROS measurements

Mitochondrial membrane potential was assessed by labeling the cells with 100 nM tetramethylrhodamine, ethyl ester, perchlorate (TMRE) (Molecular Probes) for 35 min. Cells were analyzed for reactive oxygen species (ROS) production by labeling with 10 µM dihydroethidium (DHE) (Molecular Probes) for 40 min. Fluorescence of both dyes were analyzed on a FACSCalibur flow cytometer (Becton Dickinson). 10,000 events were collected for each sample. Mitochondrial membrane potential and ROS levels were expressed as mean fluorescence intensity (MFI), which was calculated by WinList software (Verity Software House).

### Apoptosis measurement

The apoptotic fraction of cells was determined by measuring the percent of cells that had subdiploid DNA content (SubG1 Fraction). Cells were harvested, fixed with cold 70% ethanol, and washed with 0.5% BSA (Sigma). DNA was stained with propdium iodide (Sigma) in 0.2% sodium citrate (Sigma), 0.2 mg/ml RNase A (Sigma), and 0.2% NP-40 (Sigma) for 30 min at 4°C in the dark. DNA content was read on the FL2 channel of a FACSCalibur flow cytometer. 50,000 events were collected for each sample.

### Matrigel invasion

*In vitro* invasion was measured using Boyden matrigel chambers (Becton Dickinson, Franklin Lakes, NJ). Cells were serum starved for 4 h prior to harvesting. Cells were plated in the upper chamber with DMEM containing 0.1% BSA, while NIH-3T3 conditioned media containing 10% FBS was used as a chemoattractant in the lower chamber. Cells were incubated for 24 h and stained with the Diff-Quik Stain kit (Dade Behring, Newark, DE).

## Results

### Mitochondrial oxidative phosphorylation defects in breast cancer cells

To determine the defect in oxidative phosphorylation in breast cancer cells we measured the changes in gene expression associated with mtOXPHOS complexes. We also measured the enzymatic activities of the various complexes. Functional analysis was performed by determining the mitochondrial membrane potential and intracellular ROS levels. MCF12A cell line, an immortalized, non-transformed cell line, was used as a control. Breast cancer cell lines demonstrating different levels of tumorgenicity were used for analysis. These cell lines include MCF7, T47D, SkBR3, and MDA-MB-231.

**Figure 1:**
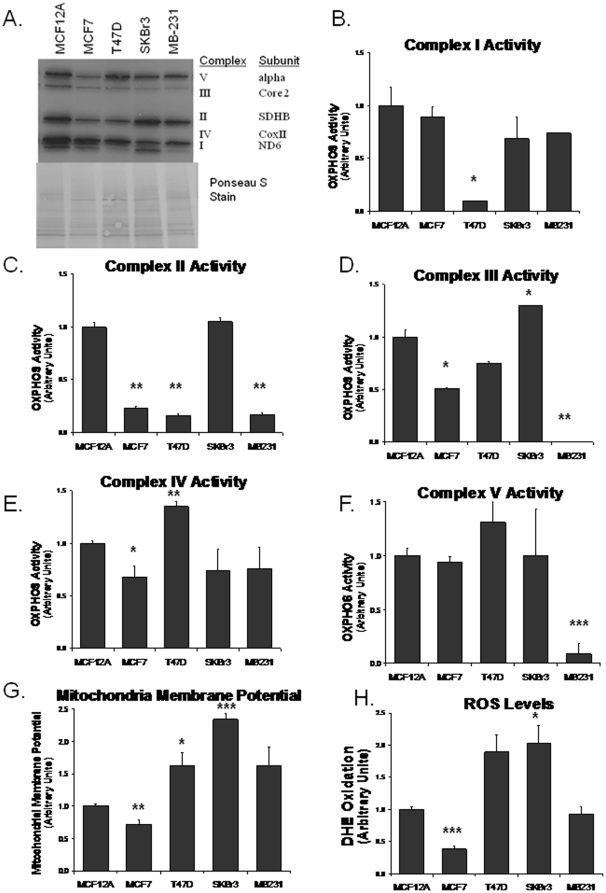
Changes in OXPHOS subunit gene expression and activity in breast cancer cells. **A.** Western blot analysis of a representative subunit from each complex. Ponceau S staining serves as a loading control. Oxidative phosphorylation enzyme activities were measured on isolated mitochondrial protein. **B**. Complex I activity was measured as the rotenone inhabitable rate of NADH oxidation. **C.** Complex II activity was measured by the succinate induced rate of reduction of DCIP. **D.** Complex III activity was measured as the rate reduction of cytochrome *c* (III) when stimulated with CoQ_2_H_2_. **E.** Complex IV activity was measured as the rate of cytochrome *c* (II) oxidation. **F.** Complex V activity was measured by the oxidation of NADH in the presence of pyruvate kinase/lactic dehydrogenase and PEP. **G.** Mitochondrial membrane potential was measured by TMRE fluorescence. **H.** Intracellular ROS was measured by DHE oxidation. Data are expressed as the mean ratio to MCF12A + 1 SEM, * *p*<0.05, ** *p*<0.005, *** *p*<0.0005.

Our study demonstrates a decreased expression of subunits of mtOXPHOS complexes in breast cancer cells as assessed by western blot (Figure 1A). MCF7 cells show a decrease in Complexes II, III and V; T47D cells have a decrease in expression of Complexes I and III; SKBr3 cells show a decrease in expression of Complex III, IV and V; MDA-MB-231 has a decrease in Complexes I, III, IV and V. The observed decrease in gene expression of mtOXPHOS subunits correlates with a decrease in individual complex activities (Figure 1B-F). Complex I activity is decreased about 5-fold in T47D cells but only about 20% in SKBr3 and MDA-MDA-MB-231 cells (Figure 1B). Complex II activity is decreased in MCF7, T47D, and MB231 cells (Figure 1C). Complex III activity is decreased in MCF7 and T47D cells, increased in SKBr3 cells, and non-detectable in MDA-MB-231 cells (Figure 1D). Complex IV activity is significantly decreased in MCF7, SKBr3, and MDA-MB-231 cells, and slightly increased in T47D. Complex V activity is increased in T47D cells and in contrast complex IV activity is decreased more than 5-fold in MDA-MB-231 cells. Interestingly, MDA-MB-231 cell line which is the most aggressive breast cancer cell line harbored the most mtOXPHOS defect compared to MCF12A cells. Except for MCF7 all other cell lines higher mitochondrial membrane potential (Figure 1G). ROS levels as measured by DHE oxidation was about two fold less in MCF7 cells while ROS level in SKBr3 and T47D cells was increased when compared to MCF12A (Figure 1H). Together these data suggest that mtOXPHOS defect is a common and consistent feature of breast cancer cells.

### Changes in Complex III subunit expression in breast cancer

**Figure 2:**
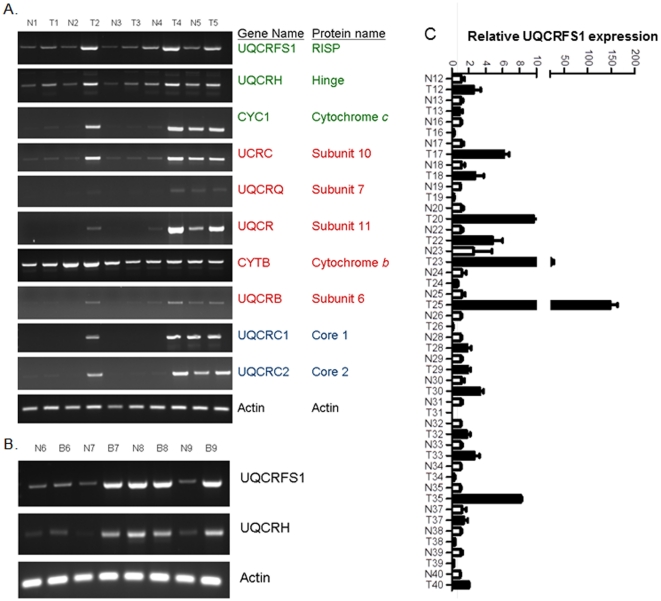
Altered Complex III gene expression in matched normal and tumor human breast tumors. **A.** *UQCRFS1*-Ubiquinol-cytochrome c reductase, Rieske iron-sulfur polypeptide 1; *UQCRH*-Ubiquinol-cytochrome c reductase hinge protein; *CYC1*-Cytochrome c-1; *UCRC*-Ubiquinol-cytochrome c reductase subunit X.; *UQCRQ*-Ubiquinol-cytochrome c reductase subunit VII; *UQCR*-Ubiquinol-cytochrome c reductase subunit XI; *CYTB*-Cytochrome b; *UQCRB*-Ubiquinol-cytochrome c reductase binding protein; *UQCRC1*-Ubiquinol-cytochrome c reductase core protein I; *UQCRC2*-Ubiquinol-cytochrome c reductase core protein II. Actin serves as a loading control. Subunits labeled in green are located in the intermembrane space, subunits labeled in red are transmemebrane, and subunits labeled in blue are located in the matrix. **B.** Coregulation of *UQCRFS1* and *UQCRH* expression in matched normal and tumor breast tissue. **C.** Quantitative RT-PCR analyses of UQCRFS1 expression in matched normal and tumor breast tissue.

Above study identified a dramatically reduced level of Complex III activity in aggressive breast cancer cell line MDA-MB-231. We therefore focused on analysis of Complex III in primary breast tumors. Complex III is a 248 kDa protein complex that comprised of 11 subunits from 10 gene products. The 11^th^ subunit is comprised of the mitochondrial targeting sequence of the RISP protein which is cleaved and inserted into the transmembrane domain of Complex III. By RT-PCR we measured expression of the 10 subunits in 5 sets of matched normal and primary breast tumors derived from same patient (Figure 2A). In all 5 cases, *UQCRFS1* (RISP protein) gene expression was higher in the breast tumor when compared to its normal breast tissue counterpart. Importantly, similar pattern of gene expression was noticed in case of *UQCRH* (encoding hinge protein). With the exception of tumor sample T2 and T4, there was no difference in expression of *CYC1, UCRC, UQCRQ, UQCR, CYTB*, *UQCRB*, *UQCRC1 UQCRC2* and *UQCRQ* in the original 5 sets (N1T1 to N5T5, Figure 2A). Additional breast tumor analyses reveal that the *UQCRFS1* and *UQCRH* genes were overexpressed. A total of 8 out of 9 breast tumors show coordinate regulation of UQCRFS1 and UQCRH suggesting a common factor involved in transcriptional regulation of these two genes.

**Figure 3:**
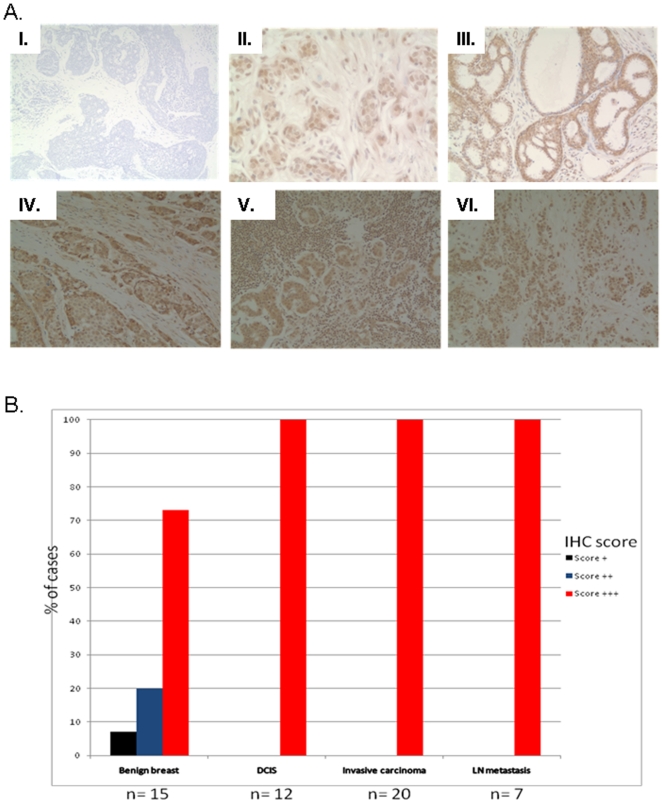
UQCRFS1/RISP expression in primary breast tumors. **A.** Representative images of IHC performed on a breast tissue array (I. Benign breast tissue incubated with secondary antibody used as a negative control; II. Benign breast tissue with no carcinoma; III. Ductal carcinoma in situ (DCIS); IV. Breast invasive ductal carcinoma (IDC); V. Invasive lobular carcinoma (ILC); VI. Metastatic breast adenocarcinoma in lymph nodes (LNM)). IHC was done with an anti-RISP antibody and visualized using DAB with hematoxylin counterstain. **B.** Graph representing semi-quantitative scoring of immunoreactivity for RISP expression in benign breast, ductal carcinoma in situ (DCIS), invasive carcinomas and metastatic breast carcinoma in lymph nodes (LN metastasis). IHC analysis was done on tissue array containing 54 breast tissue cores with anti-RISP antibody. Note high expression (score +++) of RISP protein in DCIS, invasive primary tumor, and metastatic breast carcinoma compared to benign breast tissue. RISP protein was visualized using DAB with hematoxylin counterstain.

The RISP subunit (encoded by *UQCRFS1*) is an iron-sulfur protein that accepts electrons from ubiquinol and transfers them to cytochrome *c*. This reaction acts as the rate limiting step for electron flow through Complex III. Because *UQCRFS1* was overexpressed in small set (9 samples), we quantified its expression in an additional 40 breast tumors. Figure 2C demonstrate that *UQCRFS1* was overexpressed in 14 out 40 (35%) samples. We also measured *UQCRFS1* expression at protein level. Using the tissue microarrays with several breast core biopsies from different cases, we screened for RISP protein expression in a relatively large number of cases under the same experimental conditions (Figure 3A and B). RISP was mildly expressed (score +) in one case, moderately expressed (score ++) in 3 cases and highly expressed (score +++) in 2 cases of benign breast tissue from subjects without breast carcinoma. Examination of the breast tumor sections revealed that 100% of benign breast carcinomas, ductal carcinoma in situ (DCIS*)*, invasive ductal carcinoma (IDC), invasive lobular carcinoma (ILC) and lymph node metastases (LNM) have high expression of RISP. Interestingly, RISP expression in benign glands is lower when compared to matched DCIS cells in cases containing both components in the same tissue core. Negative control sections incubated with secondary antibody showed no reaction. We conclude that RISP is overexpressed in significant number of breast tumors and that its expression does not correlate with tumor grade.

### UQCRFS1 and UQCRH over expression in variety of tumor types

**Figure 4:**
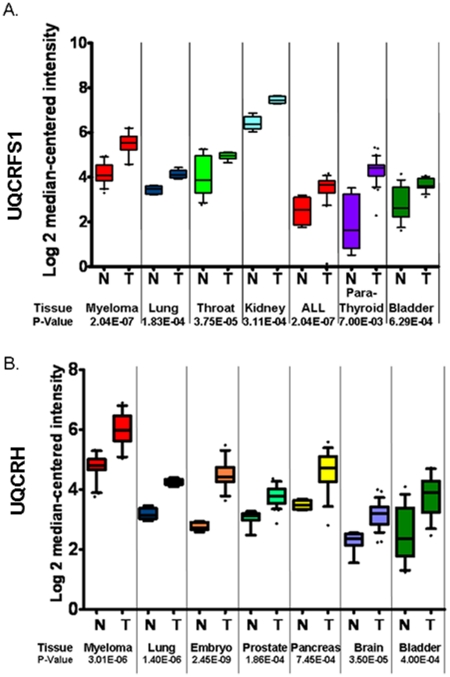
Altered Complex III expression in variety of tumors. Oncomine database analysis of *UQCRFS1* (A) and *UCQRH* (B) upregulation in normal (N) versus tumor (T) human tissue. **A.** Increased *UQCRFS1* expression in blood (myeloma) [18], lung (squamous cell lung carcinoma) [19], throat (oropharyngeal carcinoma) [20], kidney (renal oncocytoma) [21], blood (B-cell ALL) [22], parathyroid (parathyroid gland adenoma) [23], and bladder (infiltrating bladder urothelial carcinoma) [24]. **B.** Increased *UQCRH* expression in blood (myeloma) [18], lung (squamous cell lung carcinoma) [19], embryo (seminoma) [25], prostate (prostate adenocarcinoma) [26], pancreas (pancreatic adenocarcinoma) [27], brain (glioblastoma) [28], and bladder (infiltrating bladder urothelial carcinoma) [24].

The above study suggests impaired regulation of RISP and Hinze Complex III subunits in breast tumors. We determined if expression of *UQCRFS1* (encoding RISP protein) and *UQCRH* (encoding Hinge) was also increased in other cancers. Figure 4 demonstrates over expression of *UQCRFS1* in blood (myeloma) [18], lung (squamous cell lung carcinoma) [19], throat (oropharyngeal carcinoma) [20], kidney (renal oncocytoma) [21], blood (B-cell ALL) [22], parathyroid (parathyroid gland adenoma) [23], and bladder (infiltrating bladder urothelial) carcinomas [24]. Our study also revealed increased expression of *UQCRH* in myeloma [18], lung tumors [19], embryo (seminoma) [25], prostate tumors [26], pancreatic tumors [27], brain (glioblastoma) [28], and bladder (infiltrating bladder urothelial) carcinoma [24]. We conclude that Complex III subunits encoded by *UQCRFS1* and *UQCRH* are overexpressed in a variety of primary tumors derived from different organs and that *UQCRFS1* and *UQCRH* regulated in myeloma, lung and bladder cancers.

### RISP knock-down inhibits matrigel invasion of breast cancer cells

**Figure 5:**
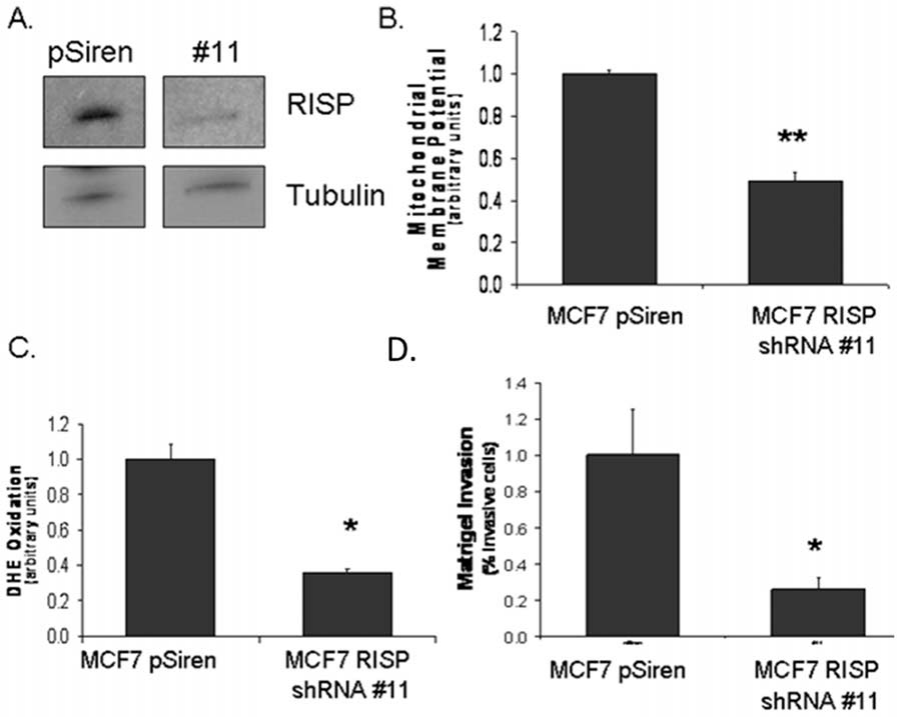
UQCRFS1/RISP knock-down results in mitochondrial dysfunction. **A.** RISP levels in MCF7 cells 3 day after transduction with anti-RISP shRNA (clone #11). pSiren was used as the vector control. **B.** Mitochondrial membrane potential of RISP knockdown cells. Membrane potential was measured by TMRE fluorescence. **C.** ROS levels in RISP knockdown cells. ROS was measured by oxidation of DHE. **E.** Invasion in RISP knockdown cells. Invasion was measured by a Matrigel invasion assay. Data represent ratio to MCF7 + 1 SEM, * *p*<0.05, ** *p*<0.005.

In order to define RISP associated mitochondrial function and its role in breast cancer, we knockdown this protein in MCF7, a RISP overexpressing cell line. When MCF7 cells were transiently transduced with shRNA directed towards UQCRFS1, we observed a 75% decrease in RISP expression (Figure 5A). RISP knockdown led to decrease in mitochondrial membrane potential when compared to the control (Figure 5B). This trend is also observed in ROS production (Figure 5C). These results suggest that RISP is essential for maintaining proper mitochondrial function. To determine if a reduced RISP expression results in reduced tumor promoting properties we measured invasion of matrigel *in vitro* in a Boyden chamber. There was a significant decrease in matrigel invasion in RISP knockdown MCF7 cells (Figure 5D). These studies suggest that RISP contributes to the invasive potential of breast cancer cells.

### RISP knock-down inhibits matrigel invasion of other transformed cells

**Figure 6:**
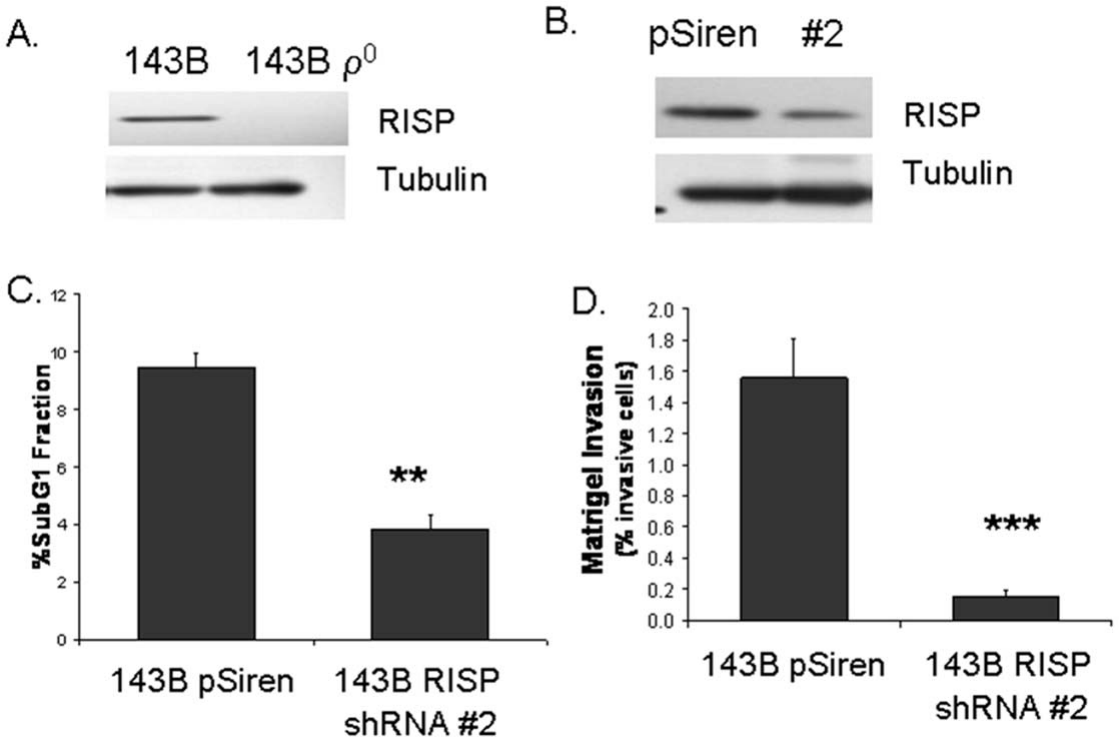
Lack of UQCRFS1/RISP expression in ρ^0^ cells. **A.** Western blot analysis of RISP expression in 143B and 143B ρ^0^ osteosarcoma cells. Tubulin served as a loading control. **B.** RISP knock-down in 143B cells transduced with either pSiren (control) or shRNA directed against RISP (clone #2). **C.** Resistance to apoptosis was measured by analyzing SubG1 fraction by DNA staining with propidium iodide. **D**. *In vitro* invasion was measured by a Matrigel invasion assay in RISP knockdown cells. ** *p*<0.005 *** *p*<0.0005.

The above study shows RISP regulates mitochondrial function. We asked whether mitochondrial dysfunction effects RISP expression. We determined RISP expression in osteosarcoma cells (143B) and it derivative cell line devoid of mitochondrial DNA (ρ^0^ cells). RISP protein was undetected in 143B cells devoid of mtDNA (Figure 6A). To determine if the RISP knockdown in osteosarcoma cells recapitulate the effect we saw in breast cancer cells we used 143B cells (Figure 6B). A decrease in RISP expression in 143B cells led to a significant increase in resistance to apoptosis (Figure 6C). There was also a decrease in *in vitro* invasion as measured by a Matrigel invasion assay (Figure 6D). We conclude that RISP plays an important role in development of other tumors.

### RISP regulates NADPH oxidase expression

**Figure 7:**
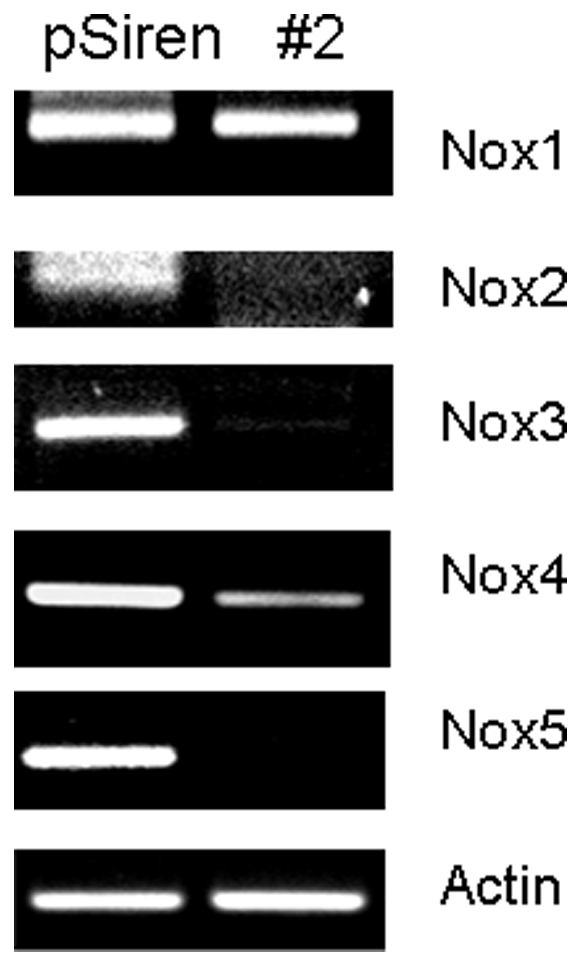
UQCRFS1/RISP knock-down decreases expression of NADPH oxidase (NOX) genes. RT-PCR analyses of NOX genes in RISP knockdown cells. Actin served as a loading control.

Above study suggest that RISP expression is associated with production of ROS. NADPH oxidase (NOX) proteins are well known producers of ROS in cells. So we determined whether RISP associated ROS production was mediated by NOX proteins. Figure 7 shows that RISP knockdown leads to decrease in expression of *NOX2, NOX3, NOX4*, and *NOX5*. Interestingly, *NOX1* expression was unchanged. This study suggests that a defect in Complex III influences expression of ROS producing NOX proteins.

## Discussion

In this study we examined the OXPHOS function in breast cancer cells. The activity of mtOXPHOS complexes III was reduced significantly in the metastatic and aggressive breast cancer cell lines. Detailed examination of Complex III showed that the iron-sulfur RISP protein, a vital part of electron transfer through Complex III, is up-regulated in human breast tumors (Figure 2). To determine RISP is causally related to breast tumorigenesis, we used shRNA against RISP to knock-down its expression. This resulted in a decrease in invasiveness in breast (MCF7) and osteosarcoma (143B) cells as measured by the matrigel invasion assay (Figure 5D and 6D). Our study is supported by previous report suggesting a decrease in matrigel invasion due to defect in OXPHOS complex [29].

Knock-down of RISP protein caused altered mitochondrial function (Figure 5). Electron transfer to the iron-sulfur cluster in RISP is the rate-determining step in Complex III activity [9]. Previous experiments have shown that Complex III proteins that are lacking the Fe-S cluster in the RISP subunit have an open proton channel through the complex allowing for the unregulated translocation of protons [30], [31]. This could potentially explain the decrease in mitochondrial membrane potential seen when RISP is knocked-down in MCF7 cells. RISP protein was absent in cells devoid of mitochondrial DNA (Figure 6A). Our previous study suggest that the core 1 subunit (*UQCRQ1*) expression of Complex III was decreased in cells devoid of mtDNA, however, when mtDNA was replaced in these cells the expression levels of the core 1 subunit recovered back to the level of wild-type cells [32].

Consistent with our study showing overexpression of RISP in breast tumors and other tumors, previous studies report genetic amplification of the UQCRFS1/RISP gene not only in breast but also in ovarian cancers and in leukemia [33]–[35]. UQCRFS1/RISP amplification was found to be in 12.8% of breast tumors examined by Ohashi *et al*. [35] and 13.6% ovarian cases reported by Kaneko *et al*. [34]. Data presented in this study suggest a correlation between the mtOXPHOS defects with the severity of breast cancer in that the aggressive metastatic breast cancer cell line, MDA-MB-231, contained the most aberrations in mtOXPHOS activity. Indeed, Complex III activity was undetected and complex V activity was reduced to 10% in MDA-MB-231. It is noteworthy that MDA-MB-231 is a triple negative (ER-, PR- and HER2-) cell line demonstrated to form highly aggressive breast tumors. These data suggest that impaired Complex III is involved in development of breast cancer and that aggressiveness of breast cancer is associated with type and nature of mtOXPHOS defect.

The mitochondria and NOX family proteins are major sources of cellular ROS in the cell. NOX proteins are a superoxide producing family of proteins that traditionally have been thought to serve as host-defense [36]. Recent studies have shown that NOX proteins are also expressed in a variety of tissues and play a role in cellular signaling and tumorigenesis through ROS production [15], [37], [38]. We has previously shown that the NOX proteins are regulated by mitochondrial dysfunction [16] and are also associated with breast cancer [15]. Analyses of NOX in RISP knockdown cells revealed decreased level of NOX expression coinciding with decreased matrigel invasion (Figure 6). These study suggest NOX mediated ROS may play a role in a key step of breast tumoroigenesis. In summary, our data suggest that an impaired Complex III function contributes to the development of breast cancer.
